# Supplementary material for: RNF20 links the DNA damage response and metabolic rewiring in lung cancer through HIF1α
Source: Nat Commun. 2025 May 28;16:4929. doi: 10.1038/s41467-025-60223-4 (PMC12120090; doi:10.1038/s41467-025-60223-4)
Supplement: Supplementary file 1 — Supplementary Information [file 41467_2025_60223_MOESM1_ESM.pdf]

Supplementary Information

for

RNF20 links the DNA damage response and metabolic rewiring in lung cancer through HIF1α

Hao Liu[1§], Yongqin Tang[1§], Anshu Singh[1,2§], Joaquim Vong[2], Julio Cordero[1], Arthur Mathes[1], Rui Gao[1,2], Yanhan Jia[1,2], Boyan K. Garvalov[3], Till Acker[4], Gernot Poschet[5], Rüdiger Hell[5], Marc A. Schneider[6,7], Joerg Heineke[8,9], Thomas Wieland[9,10], Guillermo Barreto[11], Adelheid Cerwenka[12,13], Michael Potente[2,14,15], Sofia-Iris Bibli[16,17], Rajkumar Savai[18,19], and Gergana Dobreva[1,2,16,20*]

*Corresponding author, email: Gergana.Dobreva@medma.uni-heidelberg.de

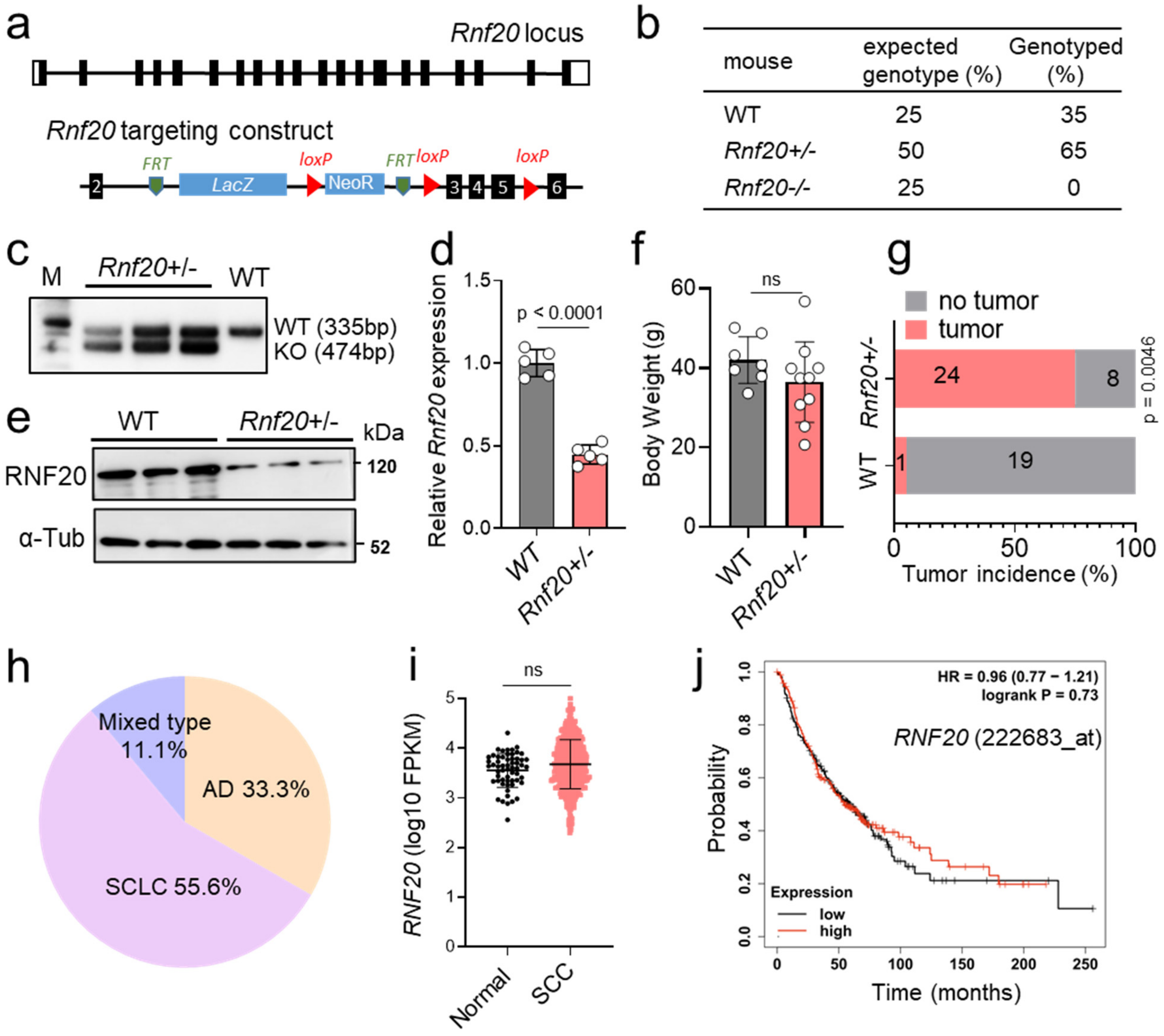

Supplementary Fig. 1. *Rnf20* loss in lung cancer. a Schematic diagram of the *Rnf20* genomic locus and the *Rnf20* targeting construct[33]. **b** Percentage distribution of expected genotypes compared to the actual genotypes of mice obtained after breeding. **c** PCR genotyping results showing distinct bands for wild-type (WT) and knockout (KO) alleles. **d** mRNA expression of *Rnf20* in lungs of control (WT) and *Rnf20+/-* mice (n = 5). **e** WB analysis of protein extracts from lungs of WT and *Rnf20+/-* mice using an anti-RNF20 antibody. **f** Body weight of WT (n = 7) and *Rnf20+/-* (n = 10) mice at 1 year of age. **g** Bar plot presenting the number of WT and *Rnf20+/-* mice with or without tumors. **h** Pie chart showing the percentage of AD, SCLC or mixed type tumors (with characteristics of both AD and SCLC) in *Rnf20+/-* tumor-bearing mice. **i** Normalized expression of *RNF20* in normal lung (n = 58) and lung squamous cell carcinoma (SCC) (n = 502) tissues in TCGA datasets. FPKM stands for

fragments per kilobase of exon per million mapped fragments. **j** Overall survival (Kaplan-Meier plot) of lung squamous cell carcinoma patients[34] expressing high vs low levels of *RNF20*. For pairwise comparions, statistical analysis was performed using two-tailed Student’s t-test (**d**, **f**, **i**). Data are shown as mean ± SEM. ns, non-significant. 'n' indicates biological replicates. Source data are provided as a Source Data file.

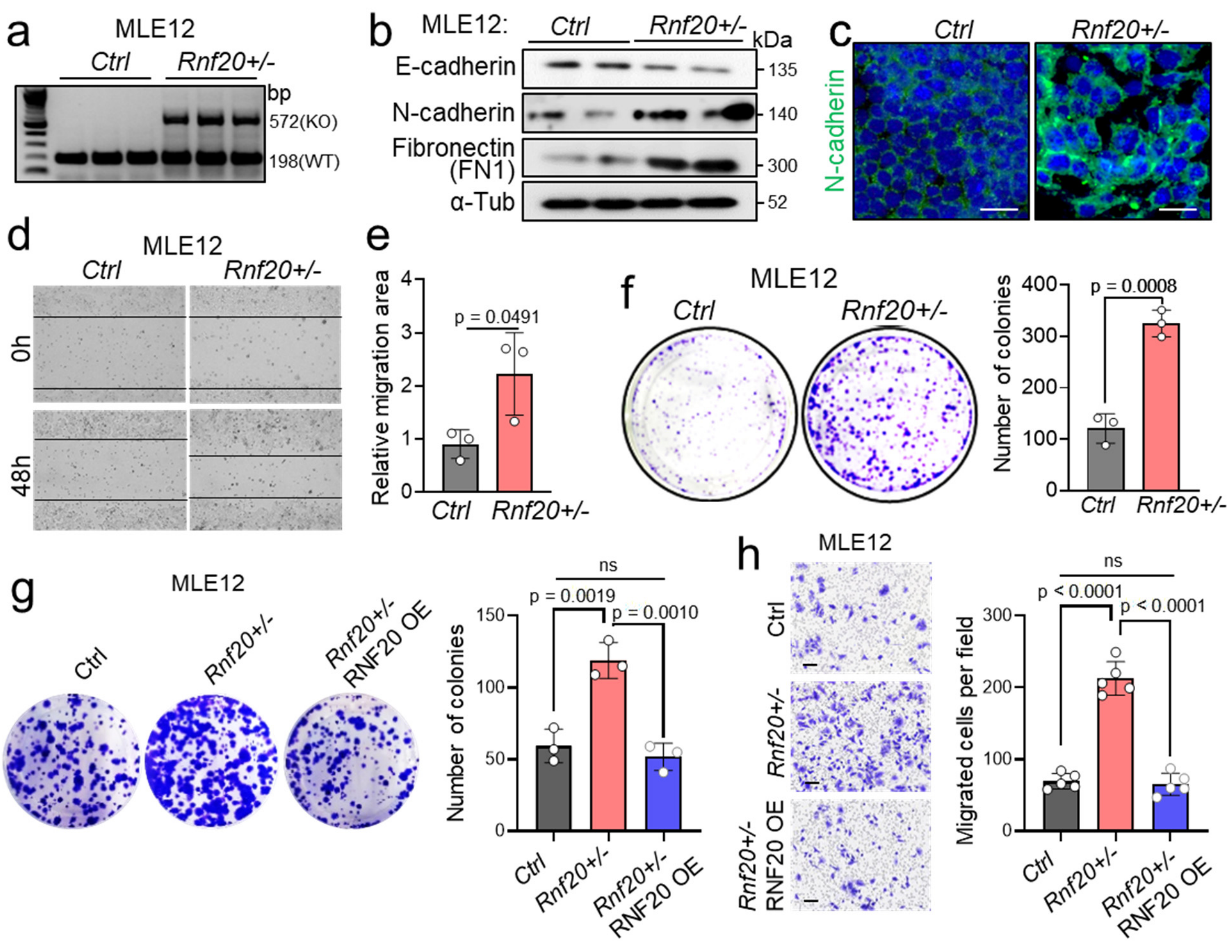

Supplementary Fig. 2. *Rnf20* haploinsufficiency induces cell migration and anchorage-independent growth. a PCR genotyping results showing distinct bands for WT and *Rnf20* KO alleles in control and *Rnf20+/-* MLE12 cells. **b** Western blot analysis of total protein extracts of control and *Rnf20+/-* MLE12 cells using E-cadherin, N-cadherin and fibronectin antibodies. α-Tubulin served as a loading control. **c** Immunostaining for N-cadherin of control and *Rnf20+/-* MLE12 cells. Scale bars, 20 µm. **d, e** Representative images of wound gap closure (**d**) and quantification of the relative migration area (**e**) in a scratch wound healing assay with control and *Rnf20+/-* MLE12 cells (n = 3). Scale bars, 20 µm. **f** Clonogenic assay performed with control and *Rnf20+/-* MLE12 cells. Repesentative images are shown on the left, quantification of the number of colonies is shown on the right (n = 3). **g** Clonogenic assay performed with control, *Rnf20+/-* and RNF20 overexpressing (OE) *Rnf20+/-* MLE12 cells. Repesentative images are shown on the left, quantification of the number of colonies is shown on the right (n = 3). **h** Boyden chamber migration assay with control, *Rnf20+/-* and RNF20 overexpressing (OE) *Rnf20+/-* MLE12 cells (left) and quantification of the number of migrated cells per field

(right) (n = 5). Statistical analysis in (**e**, **f**) was performed using a two-tailed Student's t-test. Multiple comparisons in (**g**, **h**) were performed using one-way ANOVA with Turkey's multiple comparisons test. Data are shown as mean ± SEM. 'n' indicates biological replicates. Source data are provided as a Source Data file.

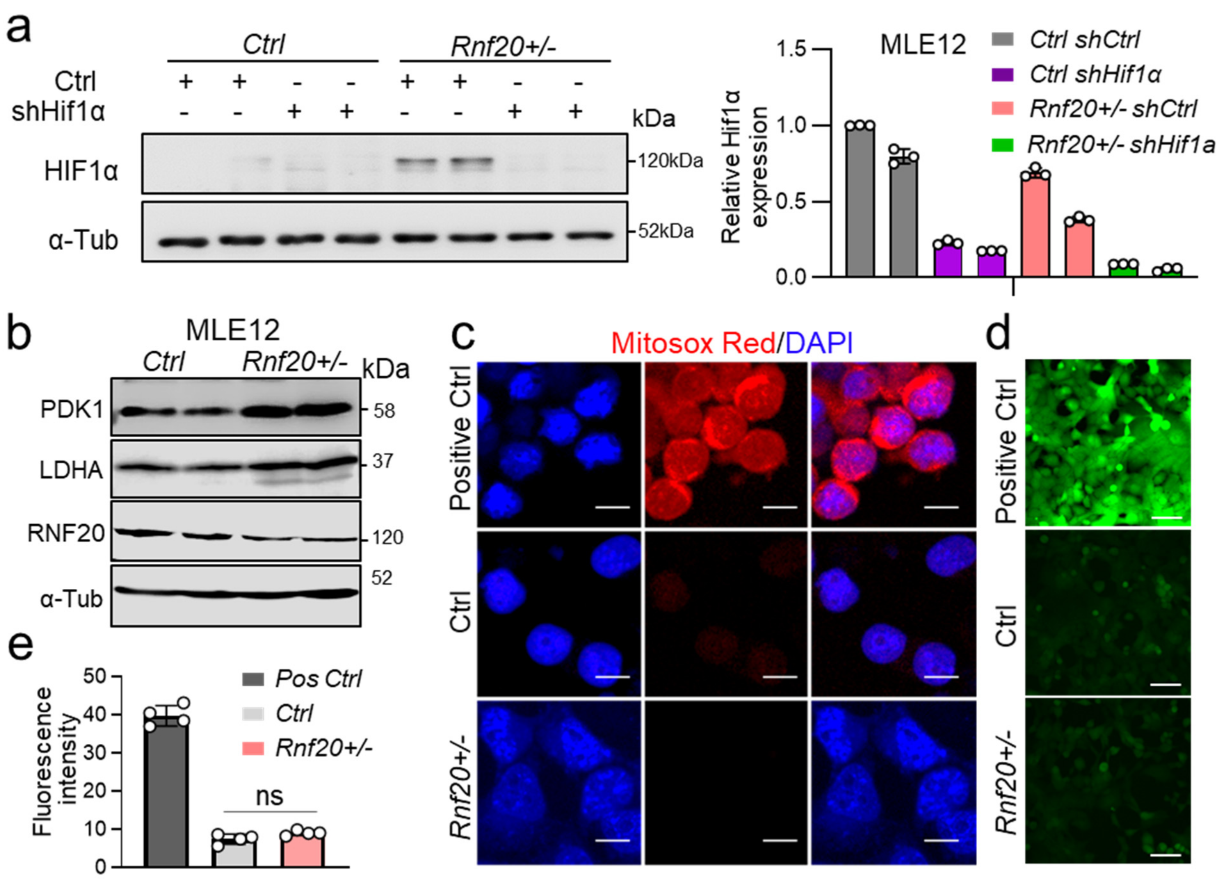

Supplementary Fig. 3. Functional and molecular alterations upon *Rnf20* loss of function. **a** Western blot analysis of total protein extracts of control and *Rnf20+/-* MLE12 cells overexpressing either control shRNA or shRNA against *Hif1a* using HIF1α antibody (left panel) and qPCR analysis of *Hif1a* mRNA expression (right panel). **b** Western blot analysis of total protein extracts of control and *Rnf20+/-* MLE12 cells using PDK1, LDHA and RNF20 antibodies. **c** Mitochondrial superoxide production in control and *Rnf20+/-* MLE12 cells measured with the mitochondrial superoxide indicator MitoSOX. Scale bars, 20 μm. **d, e** Total Reactive Oxygen Species (ROS) levels in control and *Rnf20+/-* MLE12 cells. Images of cells stained with the total Reactive Oxygen Species (ROS) Assay 488 nm kit (**d**) and fluorescence intensity measurements (**e**). Scale bars (**d**), 100 μm. Statistical analysis in (**e**) was performed using a two-tailed Student's t-test. Data are shown as mean ± SEM. ns, non-significant. 'n' indicates biological replicates. Source data are provided as a Source Data file.

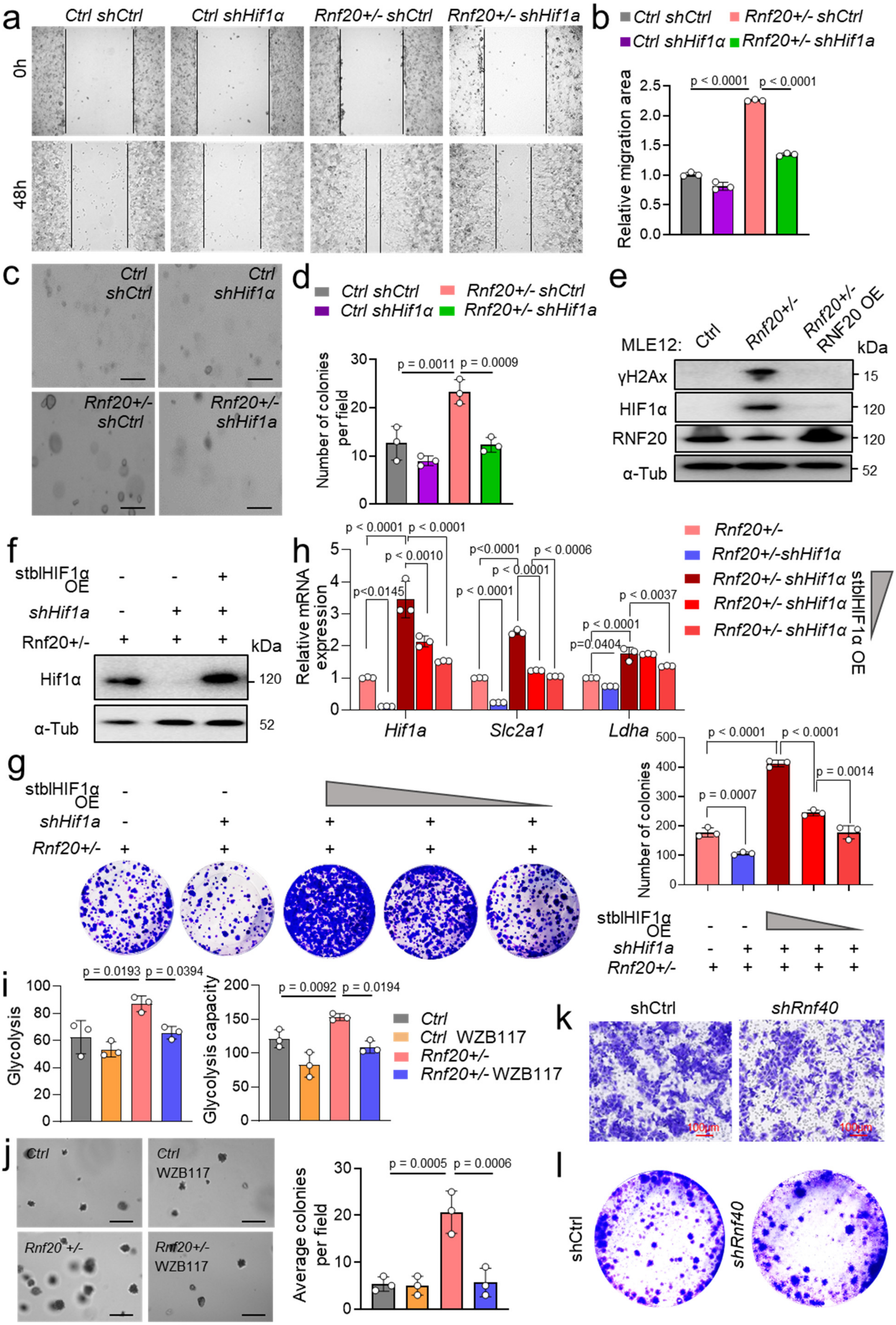
a
Ctrl shCtrl
Ctrl shHif1α
Rnf20+/- shCtrl
Rnf20+/- shHif1a
0h
48h
b
Ctrl shCtrl
Rnf20+/- shCtrl
Ctrl shHif1α
Rnf20+/- shHif1a
Relative migration area
p < 0.0001
p < 0.0001
c
d
Number of colonies per field
p = 0.0011
p = 0.0009
e
MLE12:
Ctrl
Rnf20+/-
Rnf20+/- RNF20 OE
kDa
γH2Ax
HIF1α
RNF20
α-Tub
f
stblHIF1α OE
shHif1a
Rnf20+/-
Hif1α
h
Relative mRNA expression
Hif1a
Slc2a1
Ldha
Rnf20+/-shHif1α
stblHIF1α OE
g
Number of colonies
i
Glycolysis
Glycolysis capacity
Ctrl
Ctrl WZB117
Rnf20+/-
Rnf20+/- WZB117
j
Average colonies per field
k
shCtrl
shRnf40
l

Supplementary Fig. 4. *Hif1a* silencing or inhibition of glucose uptake rescues the increased growth and migration of *Rnf20+/-* lung epithelial cells. a, b Representative images of wound gap closure **(a)** and quantification of the relative migration area **(b)** in a scratch wound healing assay with control and *Rnf20+/-* MLE12 cells overexpressing either control shRNA or shRNA against *Hif1a* (n = 3). **c, d** Soft agar assay to determine the anchorage-independent growth and quantification of the number of colonies (**d**, n = 3). Scale bars, 200 µm. **e** Western blot analysis of γH2Ax, HIF1α and RNF20 in total cell lysates from control, *Rnf20+/-* and RNF20 OE *Rnf20+/-* MLE12 cells. **f** Western blot analysis of HIF1α in total cell lysates from *Rnf20+/-* MLE12 cells, or *Rnf20+/-* cells expressing shRNA against *Hif1a* either alone or together with a constitutively active (non-degradable) human HIF1α overexpression construct (stblHIF1α OE). **g** Clonogenic assay performed with *Rnf20+/-* MLE12 cells, or *Rnf20+/-* cells expressing shRNA against *Hif1a* alone or together with varying amounts of stblHIF1α OE construct. Representative images are shown on the left, quantification of the number of colonies is shown on the right (n = 3). **h** qPCR analysis of glycolysis and hypoxia-related genes in *Rnf20+/-* MLE12 cells, or *Rnf20+/-* cells expressing shRNA against *Hif1a*, either alone or in combination with varying amounts of stblHIF1α OE construct (n = 3). **i** Relative glycolysis (left) and maximum glycolytic capacity (right) after addition of glucose and oligomycin on control and *Rnf20+/-* MLE12 cells treated either with DMSO or with WZB117 measured by Seahorse XF Glycolysis Stress Test Kit. **j** Soft agar assay to determine the anchorage-independent growth of control and *Rnf20+/-* MLE12 cells treated either with DMSO or with WZB117 (left) and quantification of the number of colonies (right) (n = 3). Scale bars, 200 µm. **k** Representative images of Boyden chamber-based migration assay with control or *Rnf40*-mediated knockdown MLE12 cells. Scale bars, 100 µm. **l** Representative images of clonogenic assay performed with control or *Rnf40*-knockdown MLE12 cells. Multiple comparisons were performed using one-way ANOVA with Šídák's (**b**, **d**, **i**, **j**) or Tukey's (**g**, **h**) multiple comparisons test. Data are shown as mean ± SEM. 'n' indicates biological replicates. Source data are provided as a Source Data file.

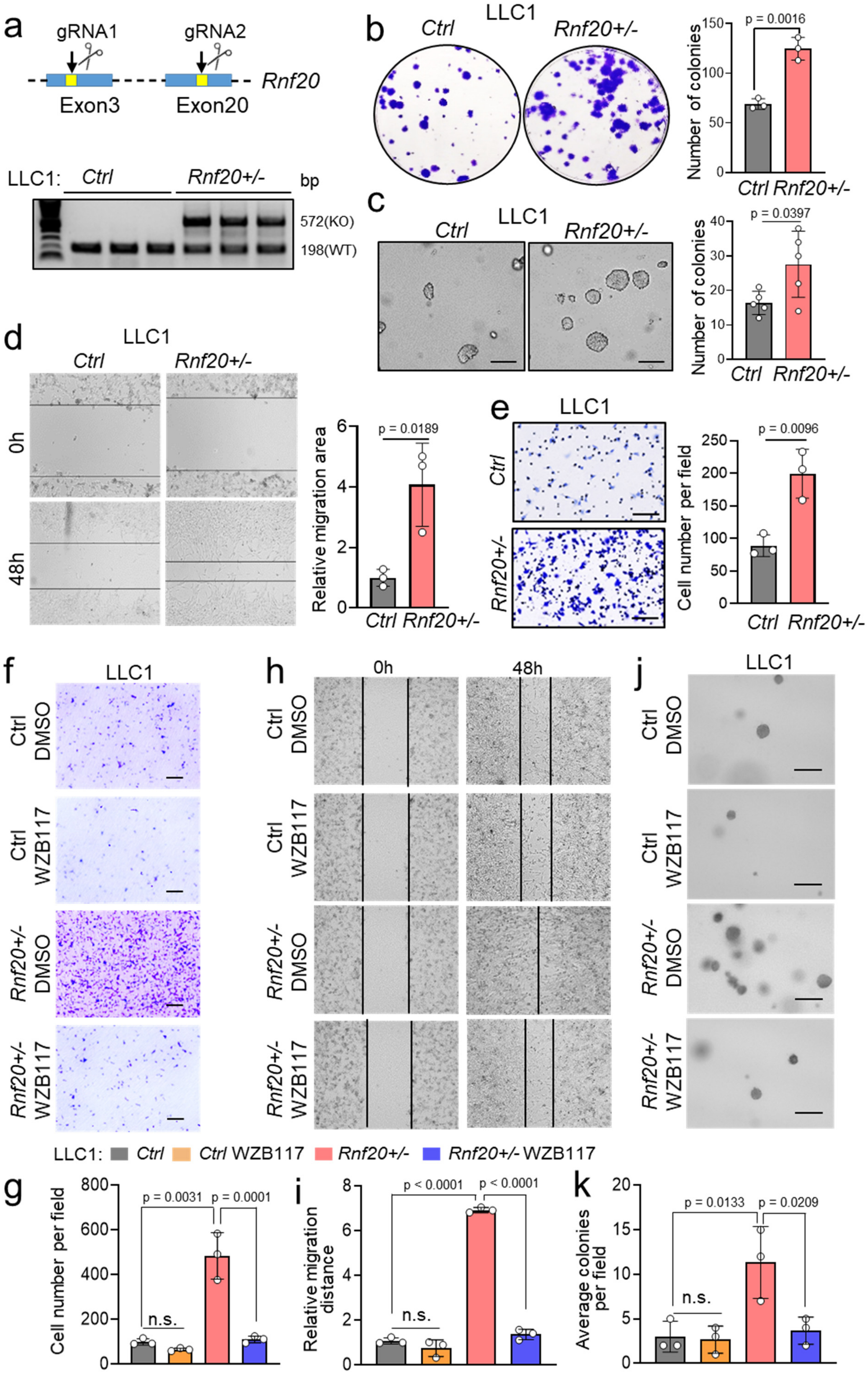
a
gRNA1
gRNA2
Rnf20
Exon3
Exon20
LLC1: Ctrl Rnf20+/-
bp
572(KO)
198(WT)
b
LLC1
Ctrl
Rnf20+/-
Number of colonies
p = 0.0016
Ctrl Rnf20+/-
c
LLC1
Ctrl
Rnf20+/-
Number of colonies
p = 0.0397
d
LLC1
Ctrl
Rnf20+/-
0h
48h
Relative migration area
p = 0.0189
e
LLC1
Ctrl
Rnf20+/-
Cell number per field
p = 0.0096
f
LLC1
Ctrl DMSO
Ctrl WZB117
Rnf20+/- DMSO
Rnf20+/- WZB117
h
0h
48h
j
LLC1
LLC1: Ctrl Ctrl WZB117 Rnf20+/- Rnf20+/- WZB117
g
Cell number per field
p = 0.0031
p = 0.0001
n.s.
i
Relative migration distance
p < 0.0001
p < 0.0001
n.s.
k
Average colonies per field
p = 0.0133
p = 0.0209
n.s.

Supplementary Fig. 5. *Hif1a* silencing or inhibition of glucose uptake rescues the increased growth and migration of *Rnf20+/-* LLC1 lung cancer cells. a Schematic representation of the strategy used to generate *Rnf20+/-* LLC1 cells (top), and PCR genotyping results showing distinct bands for WT and *Rnf20* KO alleles. The scissors icon is created in BioRender; Lityagina, O. (2025) https://BioRender.com/s41f438. **b** Reprtesentative images of a clonogenic assay with control and *Rnf20+/-* LLC1 cells (left) and quantification of the number of colonies in the soft agar (right, n = 3). **c** Reprtesentative images of a soft agar assay to determine the anchorage-independent growth of control and *Rnf20+/-* LLC1 cells (left) and quantification of the number of colonies in the soft agar (right, n = 5). Scale bars, 200 µm. **d** Representative images of wound gap closure (left) and quantification of the relative migration area (right) in a scratch wound healing assay with control and *Rnf20+/-* LLC1 cells (n = 3). **e** Boyden chamber migration assay with control and *Rnf20+/-* LLC1 cells showing representative images (left) and quantification of the number of migrated cells per field (right). n = 3. Scale bars, 150 µm. **f, g** Representative images of Boyden chamber-based migration assay with control and *Rnf20+/-* LLC1 cells treated either with DMSO or with WZB117 (**f**) and quantification on the migrated cells per field (**g,** n = 3). Scale bars, 100 µm. **h, i** Representative images of scratch wound healing assay with control and *Rnf20+/-* LLC1 cells treated either with DMSO or with WZB117 (**h**), and quantification of the relative migration area (**i**) (n = 3). **j, k** Soft agar assay to determine the anchorage-independent growth of control and *Rnf20+/-* LLC1 cells treated either with DMSO or with WZB117 (**j**) and quantification of the number of colonies (n = 3) (**k**). Scale bars, 200 µm. Statistical analysis in (**b**, **c**, **d**, **e**) was performed using a two-tailed Student's t-test. Multiple comparisons in (**g**, **i**, **k**) were performed using one-way ANOVA with Turkey's multiple comparisons test. Data are shown as mean ± SEM. 'n' indicates biological replicates. Source data are provided as a Source Data file.

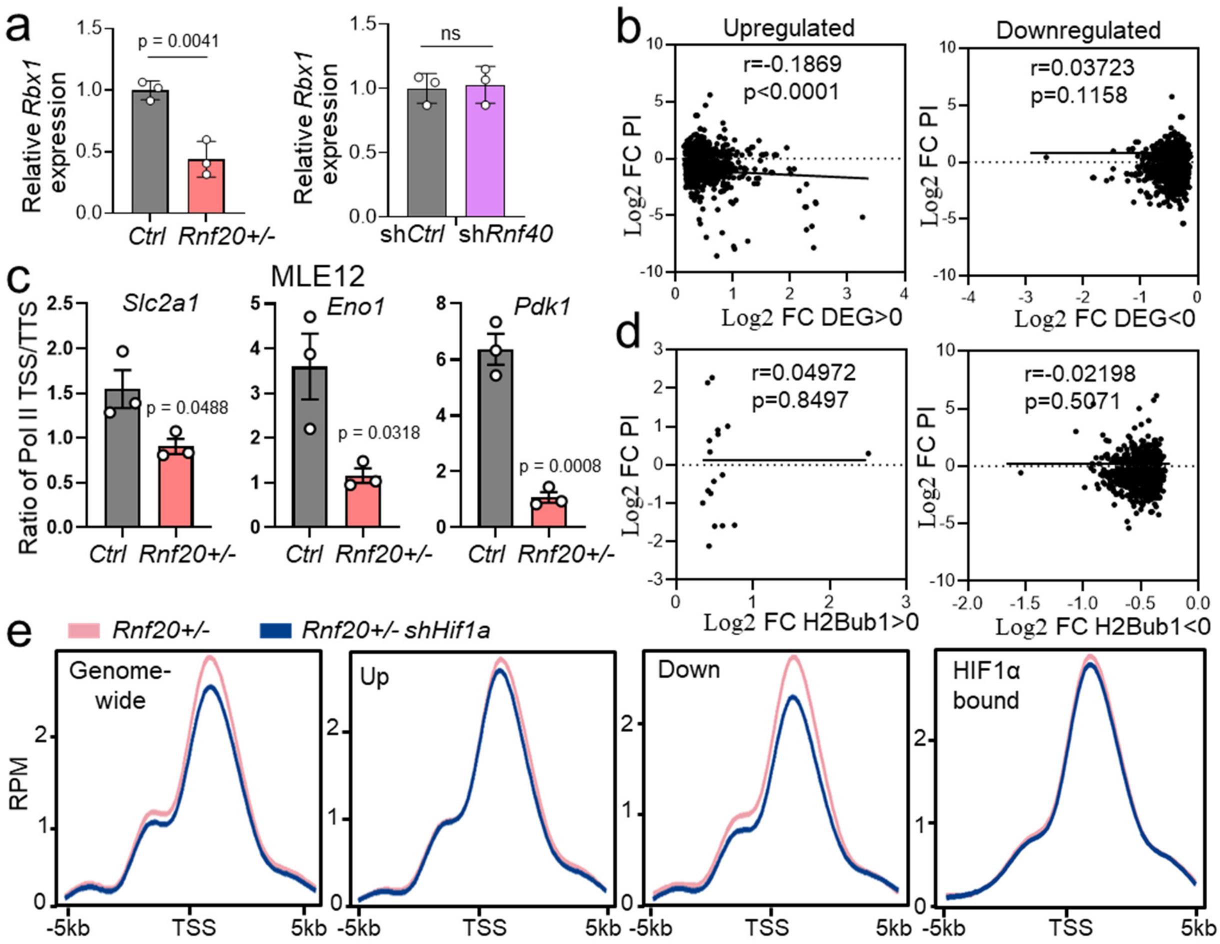

Supplementary Fig. 6. RNF20- and HIF1α-dependent transcriptional and H3K4me3 changes. a Relative *Rbx1* expression in control versus *Rnf20*+/- MLE12 cells (left), or in control and *Rnf40*-mediated knockdown MLE12 cells (right, n = 3). **b** Pearson correlation (r) for the Log2 FC of PI and Log2 FC of upregulated (left) or downregulated (right) genes in *Rnf20*+/- versus control MLE12 cells. **c** Ratio of the Pol II enrichment at the TSS and TTS of *Scl2a1*, *Eno1* and *Pdk1* in control and *Rnf20+/-* MLE12 cells determined by Pol II Chip-qPCR (n = 3). TSS, Transcription Start Site; TTS, Transcription Termination Site. **d** Pearson correlation (r) for the Log2 FC of PI and Log2 FC of genes showing increased (left) or decreased (right) H2Bub1 levels in *Rnf20*+/- versus control MLE12 cells. **e** Average genome-wide H3K4me3 ChIP-seq signal, as well as H3K4me3 ChIP-seq signal at genes that are upregulated or downregulated upon RNF20 loss or bound by HIF1α in *Rnf20+/-* MLE12 cells, or *Rnf20+/-* MLE12 cells after shRNA mediated *Hif1a* silencing. Statistical analysis in (**a**, **c**)

was performed using a two-tailed Student's t-test. Data are shown as mean ± SEM. ns, non-significant. 'n' indicates biological replicates. Source data are provided as a Source Data file.

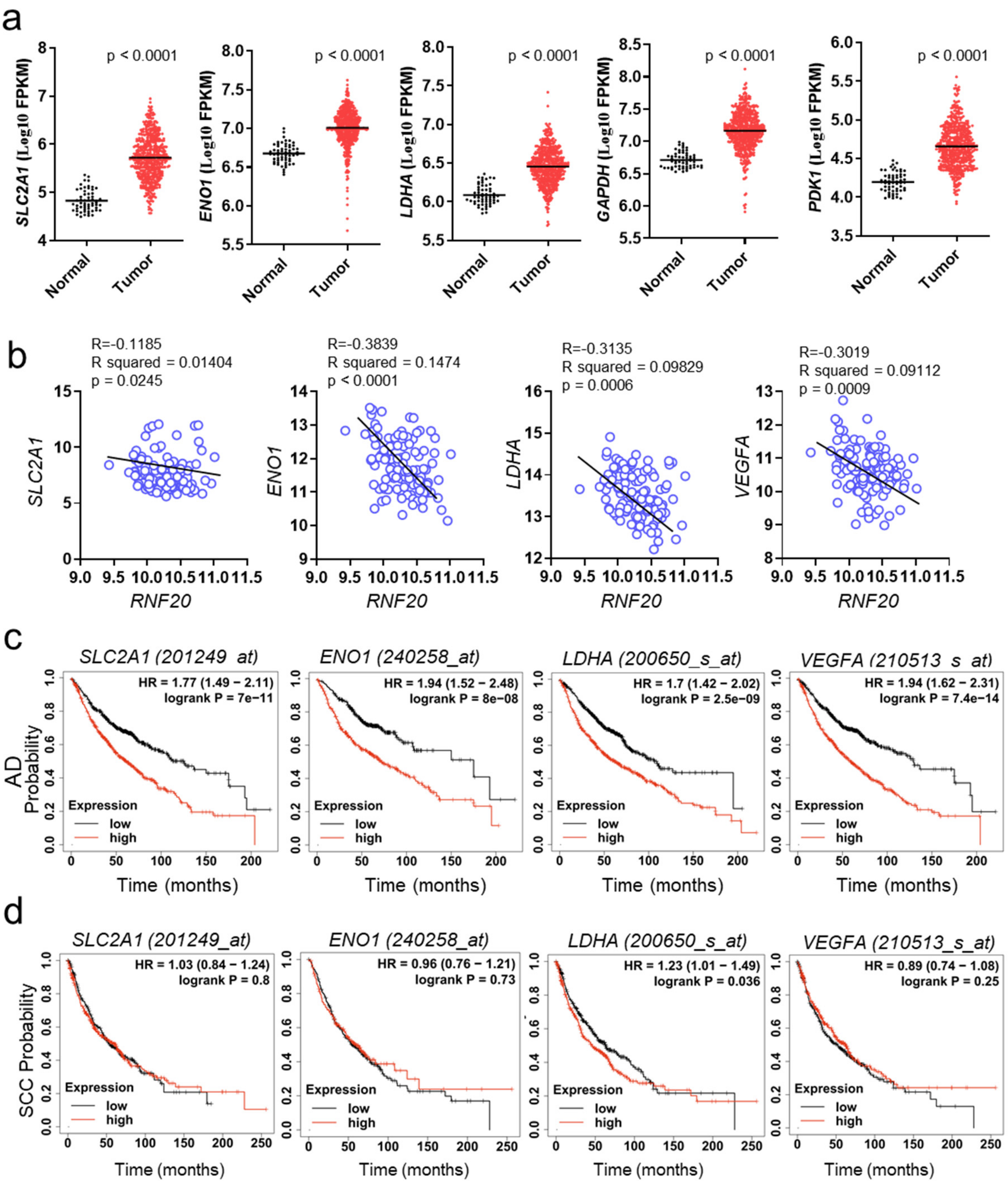

Supplementary Fig. 7 Expression levels of metabolic genes in lung AD and lack of correlation between the expression of glycolytic enzymes and survival of SCC patients. **a** Normalized expression of the metabolic enzymes *ENO1*, *PDK1*, *LDHA*, *SLC2A1* and *GAPDH* in normal lung (n = 59) and adenocarcinoma (n = 532) tissues in the TCGA datasets. **b** Pearson correlation (r) for the mRNA expression levels of *RNF20* and genes involved in glycolysis (*SLC2A1, ENO1, LDHA*) or the hypoxic response (*VEGFA*) in lung AD cancer datasets (GSE19188, GSE27262). **c** Overall survival (Kaplan-Meier plot) of lung

adenocarcinoma patients[34] expressing high vs low levels of the RNF20/HIF1α-dependent genes, *SLC2A1*, *ENO1*, *LDHA* and *VEGFA*. **d** Overall survival (Kaplan-Meier plot) of lung squamous carcinoma (SCC) patients expressing high vs low levels of *SLC2A1*, *ENO1*, *LDHA* and *VEGFA*.
