# Supplementary material for: RNF20 links the DNA damage response and metabolic rewiring in lung cancer through HIF1α
Source: Nat Commun. 2025 May 28;16:4929. doi: 10.1038/s41467-025-60223-4 (PMC12120090; doi:10.1038/s41467-025-60223-4)
Supplement: Supplementary file 2 — Description of Additional Supplementary Files [file 41467_2025_60223_MOESM2_ESM.pdf]

Description of Additional Supplementary Files

Supplementary Data 1: Gene expression values from RNA-seq of Ctrl and *Rnf20+/-* MLE12 cells and statistical differences in *Rnf20+/-* vs Ctrl MLE12 cells (n = 3 per group). Differential expression was quantified and normalized using DESeq2 (Version 1.40.0).

Supplementary Data 2: Metabolite levels in Ctrl and *Rnf20+/-* MLE12 cells measured by LC-MS/MS (n = 6 per group).

Supplementary Data 3: Pausing index values from total Pol II ChIP-seq of Ctrl and *Rnf20+/-* MLE12 cells (n = 3 per group).

Supplementary Data 4: List of genes with decreased PI in *Rnf20+/-* MLE12 cells compared to controls.

Supplementary Data 5: List of antibodies used in this study.

Supplementary Data 6: List of primers used in this study
