# Supplementary material for: RNF20 links the DNA damage response and metabolic rewiring in lung cancer through HIF1α
Source: Nat Commun. 2025 May 28;16:4929. doi: 10.1038/s41467-025-60223-4 (PMC12120090; doi:10.1038/s41467-025-60223-4)
Supplement: Supplementary file 9 — Reporting Summary [file 41467_2025_60223_MOESM9_ESM.pdf]

nature portfolio

Corresponding author(s): Gergana Dobreva

Last updated by author(s): Mar 17, 2025

Reporting Summary

Nature Portfolio wishes to improve the reproducibility of the work that we publish. This form provides structure for consistency and transparency in reporting. For further information on Nature Portfolio policies, see our Editorial Policies and the Editorial Policy Checklist.

Statistics

For all statistical analyses, confirm that the following items are present in the figure legend, table legend, main text, or Methods section.

n/a	Confirmed	
☐	☒	The exact sample size (*n*) for each experimental group/condition, given as a discrete number and unit of measurement
☐	☒	A statement on whether measurements were taken from distinct samples or whether the same sample was measured repeatedly
☐	☒	The statistical test(s) used AND whether they are one- or two-sided<br>*Only common tests should be described solely by name; describe more complex techniques in the Methods section.*
☒	☐	A description of all covariates tested
☐	☒	A description of any assumptions or corrections, such as tests of normality and adjustment for multiple comparisons
☐	☒	A full description of the statistical parameters including central tendency (e.g. means) or other basic estimates (e.g. regression coefficient) AND variation (e.g. standard deviation) or associated estimates of uncertainty (e.g. confidence intervals)
☐	☒	For null hypothesis testing, the test statistic (e.g. *F*, *t*, *r*) with confidence intervals, effect sizes, degrees of freedom and *P* value noted<br>*Give P values as exact values whenever suitable.*
☒	☐	For Bayesian analysis, information on the choice of priors and Markov chain Monte Carlo settings
☒	☐	For hierarchical and complex designs, identification of the appropriate level for tests and full reporting of outcomes
☐	☒	Estimates of effect sizes (e.g. Cohen's *d*, Pearson's *r*), indicating how they were calculated

Our web collection on statistics for biologists contains articles on many of the points above.

Software and code

Policy information about availability of computer code

Data collection	The following standard software provided by instrument suppliers was used for data collection:<br>Histology: Zeiss Axio Scan (Zeiss)<br>Western Blot (WB): Amersham Imager 600<br>Seahorse: Seahorse XFe24 Analyzer (Agilent)<br>Immunofluorescence: Zeiss LSM 710 Confocal Microscope (Zeiss)<br>Glucose, Lactate Colorimetric Assay: Tecan Spark® Multimode Microplate Reader
Data analysis	mageJ1.53q, https://imagej.nih.gov/ij/download.html<br>IGV version 2.7.0, https://software.broadinstitute.org/software/igv/download<br>STAR version 2.7.3a, https://github.com/alexdobin/STAR/blob/master/doc/STARmanual.pdf<br>BamTools version 2.5.1, https://github.com/pezmaster31/bamtools<br>Bowtie2 version 2.4.4, https://bowtie-bio.sourceforge.net/bowtie2/index.shtml<br>MultiQC version 1.9, https://multiqc.info/<br>DESeq2 version 1.40.0, http://bioconductor.org/packages/release/bioc/vignettes/DESeq2/inst/doc/DESeq2.html<br>clusterProfiler version 3.16.1, https://guangchuangyu.github.io/software/clusterProfiler/<br>SAMtools version 1.7, http://www.htslib.org/<br>Picard-tools version 1.136, https://broadinstitute.github.io/picard/<br>Bedtools version 2.30.0, https://bedtools.readthedocs.io/en/latest/<br>MiMiroot/PIC, https://github.com/MiMiroot/PIC

ggplot2 version 3.3.6, https://ggplot2.tidyverse.org/
R version 4.1.1

For manuscripts utilizing custom algorithms or software that are central to the research but not yet described in published literature, software must be made available to editors and reviewers. We strongly encourage code deposition in a community repository (e.g. GitHub). See the Nature Portfolio guidelines for submitting code & software for further information.

Data

Policy information about availability of data

All manuscripts must include a data availability statement. This statement should provide the following information, where applicable:

- Accession codes, unique identifiers, or web links for publicly available datasets
- A description of any restrictions on data availability
- For clinical datasets or third party data, please ensure that the statement adheres to our policy

Raw RNA-seq and Chip-seq data generated in this study have been deposited in GEO database under accession code GSE231317, Processed data are provided in the Supplementary Information. TCGA lung cancer data were retrieved from The Genomic Data Commons Data Portal of National Cancer Institute Center for Cancer Genomics, https://portal.gdc.cancer.gov/.

Research involving human participants, their data, or biological material

Policy information about studies with human participants or human data. See also policy information about sex, gender (identity/presentation), and sexual orientation and race, ethnicity and racism.

Reporting on sex and gender	*Use the terms sex (biological attribute) and gender (shaped by social and cultural circumstances) carefully in order to avoid confusing both terms. Indicate if findings apply to only one sex or gender; describe whether sex and gender were considered in study design; whether sex and/or gender was determined based on self-reporting or assigned and methods used. Provide in the source data disaggregated sex and gender data, where this information has been collected, and if consent has been obtained for sharing of individual-level data; provide overall numbers in this Reporting Summary. Please state if this information has not been collected. Report sex- and gender-based analyses where performed, justify reasons for lack of sex- and gender-based analysis.*
Reporting on race, ethnicity, or other socially relevant groupings	*Please specify the socially constructed or socially relevant categorization variable(s) used in your manuscript and explain why they were used. Please note that such variables should not be used as proxies for other socially constructed/relevant variables (for example, race or ethnicity should not be used as a proxy for socioeconomic status). Provide clear definitions of the relevant terms used, how they were provided (by the participants/respondents, the researchers, or third parties), and the method(s) used to classify people into the different categories (e.g. self-report, census or administrative data, social media data, etc.) Please provide details about how you controlled for confounding variables in your analyses.*
Population characteristics	*Describe the covariate-relevant population characteristics of the human research participants (e.g. age, genotypic information, past and current diagnosis and treatment categories). If you filled out the behavioural & social sciences study design questions and have nothing to add here, write "See above."*
Recruitment	*Describe how participants were recruited. Outline any potential self-selection bias or other biases that may be present and how these are likely to impact results.*
Ethics oversight	*Identify the organization(s) that approved the study protocol.*

Note that full information on the approval of the study protocol must also be provided in the manuscript.

Field-specific reporting

Please select the one below that is the best fit for your research. If you are not sure, read the appropriate sections before making your selection.

☒ Life sciences ☐ Behavioural & social sciences ☐ Ecological, evolutionary & environmental sciences

For a reference copy of the document with all sections, see nature.com/documents/nr-reporting-summary-flat.pdf

Life sciences study design

All studies must disclose on these points even when the disclosure is negative.

Sample size	No statistical measures were used to determine sample size. Sample size is based on our experience and on publications by other groups.
Data exclusions	None
Replication	All experiments were performed at least three independent times. All attempts at replication were successful.

Randomization	Mice were randomly allocated into experimental groups.
Blinding	The investigators were not blinded in regard to allocation of samples during experiments and outcome assessment. However, the outcomes were quantitative and not subjective.

Reporting for specific materials, systems and methods

We require information from authors about some types of materials, experimental systems and methods used in many studies. Here, indicate whether each material, system or method listed is relevant to your study. If you are not sure if a list item applies to your research, read the appropriate section before selecting a response.

Materials & experimental systems

n/a	Involved in the study
☐	☒ Antibodies
☐	☒ Eukaryotic cell lines
☒	☐ Palaeontology and archaeology
☐	☒ Animals and other organisms
☒	☐ Clinical data
☒	☐ Dual use research of concern
☒	☐ Plants

Methods

n/a	Involved in the study
☐	☒ ChIP-seq
☒	☐ Flow cytometry
☒	☐ MRI-based neuroimaging

Antibodies

Antibodies used

Rabbit monoclonal [EP1584Y] Anti-TTF1, Abcam, Cat#ab76013, RRID:AB_1310784, 1:150 for IF; 1:250 for IHC
Rabbit Polyclonal Anti-CD45, Abcam, Cat# ab10558, RRID:AB_442810, 1:200 for IHC
Rabbit Polyclonal Anti-Calcitonin Gene Related Peptide(CGRP), Sigma-Aldrich, Cat# C8198, RRID:AB_259091, 1:250 for IHC
Rabbit monoclonal (D8F6H) Anti-Synaptophysin, Cell Signaling Technology, Cat# 36406, RRID:AB_2799098, 1:200 for IHC
Mouse monoclonal(KCG1.1) Anti- NAPSIN A, Abcam, Cat# ab73021, RRID:AB_1269521, 1:100 for IF
Rabbit polyclonal Anti-Ki67, Abcam, Cat# AB15580, RRID:AB_805388, 1:250 for IHC
Rabbit polyclonal Anti-P63, Cell Signaling Technology, Cat# 4981, RRID:AB_2286372, 1:200 for IF
Mouse monoclonal(D6E10) Anti-RNF20, Cell Signaling Technology, Cat# 11974, RRID:AB_2797786, 1:1000 for WB, 1:200 for IHC
Mouse monoclonal Anti-alpha-Tubulin antibody, Sigma-Aldrich, Cat# T5168, RRID:AB_477579, 1:2000 for WB
Mouse monoclonal (1C12) Anti-p53, Cell Signaling Technology, Cat# 2524, RRID:AB_331743, 1:1000 for WB
Mouse monoclonal(4H1) Anti-Rb, Cell Signaling Technology, Cat# 9309, RRID:AB_823629, 1:1000 for WB
Rabbit monoclonal(20E3) Anti-Phospho-Histone H2A.X (Ser139), Cell Signaling Technology, Cat# 9718, RRID:AB_2118009, 1:1000 for WB
Rat monoclonal [DECMA-1] Anti-E Cadherin, Abcam, Cat# ab11512, RRID:AB_298118, 1:250 for IF, 1:1000 for WB
Mouse monoclonal Anti-N-Cadherin, Sigma-Aldrich, Cat# C3865, RRID:AB_262097, 1:250 for IF, 1:1000 for WB
Mouse monoclonal Anti-Fibronectin, Abcam, Cat# ab2413, RRID:AB_2262874, 1:1000 for WB
Rabbit monoclonal (D21H3) Anti-Vimentin, Cell Signaling Technology, Cat# 5741, RRID:AB_10695459, 1:250 for IF
Mouse Rabbit Anti-Glucose Transp. 1 (Glut-1), Alpha Diagnostic International, Cat# GT11-A, RRID:AB_2895172, 1:1000 for WB, 1:200 for IHC
Rabbit Polyclonal Anti-Ldha, Cell Signaling Technology, Cat# 2012, RRID:AB_2137173, 1:1000 for WB, 1:200 for IHC
Rabbit Polyclonal Anti-HIF-1α (C-Term), Cayman Chemical, Cat# 10006421, RRID:AB_409037, 1:1000 for WB
Rabbit monoclonal (D2U3T) Anti-HIF-1α, Cell Signaling Technology, Cat# 14179, RRID:AB_2622225, 1:200 for IF
Rabbit polyclonal Anti-Pdk1, Cell Signaling Technology, Cat# 3062, RRID:AB_2236832, 1:1000 for WB
Rabbit polyclonal Anti-Histone H2B, Abcam, Cat# ab1790, RRID:AB_302612, 1:1000 for WB
Rabbit monoclonal(D11) Anti-Ubiquityl-Histone H2B (Lys120), Cell Signaling Technology, Cat# 5546, RRID:AB_10693452, 1:1000 for WB
Rabbit monoclonal(D8L4Y) Anti-Rpb1, Cell Signaling Technology, Cat# 14958, RRID:AB_2687876, 1:50 for Chip
Peroxidase-AffiniPure Goat Anti-Mouse IgG (H + L) antibody, Jackson ImmunoResearch Labs, Cat# 115-035-003, RRID:AB_10015289, 1:5000 for WB
Peroxidase-AffiniPure Goat Anti-Rabbit IgG (H+L) (min X Hu Sr Prot) antibody, Jackson ImmunoResearch Labs, Cat# 111-035-045, RRID:AB_2337938, 1:5000 for WB
Goat anti-Rat IgG (H+L) Secondary Antibody, HRP, Thermo Fisher Scientific, Cat# 31470, RRID:AB_228356, 1:5000 for WB
Donkey Anti-Rabbit IgG (H+L) Antibody, Alexa Fluor 488 Conjugated, Thermo Fisher Scientific, Cat# A-21206, RRID:AB_2535792, 1:500 for IF
Donkey anti-Mouse IgG (H+L) Highly Cross-Adsorbed Secondary Antibody, Alexa Fluor 555, Thermo Fisher Scientific, Cat# A-31570, RRID:AB_2536180, 1:500 for IF
Goat anti-Rat IgG (H+L) Cross-Adsorbed Secondary Antibody, Alexa Fluor 488, Thermo Fisher Scientific, Cat# A-11006, RRID:AB_2534074, 1:500 for IF
Donkey anti-Rabbit IgG (H+L) Highly Cross-Adsorbed Secondary Antibody, Alexa Fluor 555, Thermo Fisher Scientific, Cat# A-31572, RRID:AB_162543, 1:500 for IF

Validation

All antibodies used in the study were purchased from commercial vendors and were selected because they have been validated by

the manufacture and in different publications. Validation details and relevant publications are detailed on their respective websites.
Rabbit monoclonal [EP1584Y] Anti-TTF1: https://www.abcam.com/products/primary-antibodies/ttf1-antibody-ep1584y-ab76013.html
Rabbit Polyclonal Anti-CD45: https://www.abcam.com/products/primary-antibodies/cd45-antibody-ab10558.html
Rabbit Polyclonal Anti-Calcitonin Gene Related Peptide(CGRP): https://www.sigmaaldrich.com/DE/de/search/c8198?focus=products&page=1&perpage=30&sort=relevance&term=c8198&type=product
Rabbit monoclonal (D8F6H) Anti-Synaptophysin: https://www.cellsignal.com/products/primary-antibodies/synaptophysin-d8f6h-xp-rabbit-mab/36406
Mouse monoclonal(KCG1.1) Anti- NAPSIN A: https://www.abcam.com/products/primary-antibodies/napsin-a-antibody-kcg11-ab73021.html
Rabbit polyclonal Anti-Ki67: https://www.abcam.com/products/primary-antibodies/ki67-antibody-ab15580.html
Rabbit polyclonal Anti-P63: https://www.cellsignal.com/products/primary-antibodies/phospho-p63-ser160-162-antibody/4981
Mouse monoclonal(D6E10) Anti-RNF20: https://www.cellsignal.com/products/primary-antibodies/rnf20-d6e10-xp-rabbit-mab/11974
Mouse monoclonal Anti-alpha-Tubulin antibody: https://www.sigmaaldrich.com/DE/de/search/t5168?focus=products&page=1&perpage=30&sort=relevance&term=t5168&type=product
Mouse monoclonal (1C12) Anti-p53: https://www.cellsignal.com/products/primary-antibodies/p53-1c12-mouse-mab/2524?site-search-type=Products&N=4294956287&Ntt=2524%2C&fromPage=plp&_requestid=614297
Mouse monoclonal(4H1) Anti-Rb: https://www.cellsignal.com/products/primary-antibodies/rb-4h1-mouse-mab/9309
Rabbit monoclonal(20E3) Anti-Phospho-Histone H2A.X (Ser139): https://www.cellsignal.com/products/primary-antibodies/phospho-histone-h2a-x-ser139-20e3-rabbit-mab/9718
Rat monoclonal [DECMA-1] Anti-E Cadherin: https://www.abcam.com/products/primary-antibodies/e-cadherin-antibody-decma-1-intercellular-junction-marker-ab11512.html
Mouse monoclonal Anti-N-Cadherin: https://www.sigmaaldrich.com/DE/de/product/sigma/c3865
Mouse monoclonal Anti-Fibronectin: https://www.abcam.com/products/primary-antibodies/fibronectin-antibody-ab2413.html
Rabbit monoclonal (D21H3) Anti-Vimentin: https://www.cellsignal.com/products/primary-antibodies/vimentin-d21h3-xp-rabbit-mab/5741
Mouse Rabbit Anti-Glucose Transp. 1 (Glut-1): https://www.4adi.com/4adi/rabbit-anti-mouse-glucose-transp-1-glut-1-igg-1-aff-pure-11522-p.html
Rabbit Polyclonal Anti-Ldha: https://www.cellsignal.com/products/primary-antibodies/ldha-antibody/2012
Rabbit Polyclonal Anti-HIF-1α (C-Term): https://www.caymanchem.com/product/10006421/hif-1%CE%B1-(c-term)-polyclonal-antibody
Rabbit monoclonal (D2U3T) Anti-HIF-1α: https://www.cellsignal.com/products/primary-antibodies/hif-1a-d2u3t-rabbit-mab/14179
Rabbit polyclonal Anti-Pdk1: https://www.cellsignal.com/products/primary-antibodies/pdk1-antibody/3062
Rabbit polyclonal Anti-Histone H2B: https://www.abcam.com/products/primary-antibodies/histone-h2b-antibody-chip-grade-ab1790.html
Rabbit monoclonal(D11) Anti-Ubiquityl-Histone H2B (Lys120): https://www.cellsignal.com/products/primary-antibodies/ubiquityl-histone-h2b-lys120-d11-xp-rabbit-mab/5546?site-search-type=Products&N=4294956287&Ntt=5546%2C&fromPage=plp&_requestid=616261
Rabbit monoclonal(D8L4Y) Anti-Rpb1: https://www.cellsignal.com/products/primary-antibodies/rpb1-ntd-d8l4y-rabbit-mab/14958
Peroxidase-AffiniPure Goat Anti-Mouse IgG (H + L) antibody: https://www.jacksonimmuno.com/catalog/products/115-035-003
Peroxidase-AffiniPure Goat Anti-Rabbit IgG (H+L) (min X Hu Sr Prot) antibody: https://www.jacksonimmuno.com/catalog/products/111-035-045
Goat anti-Rat IgG (H+L) Secondary Antibody: https://www.thermofisher.com/antibody/product/Goat-anti-Rat-IgG-H-L-Secondary-Antibody-Polyclonal/31470
Donkey Anti-Rabbit IgG (H+L) Antibody, Alexa Fluor 488 Conjugated: https://www.thermofisher.com/antibody/product/Donkey-anti-Rabbit-IgG-H-L-Highly-Cross-Adsorbed-Secondary-Antibody-Polyclonal/A-21206
Donkey anti-Mouse IgG (H+L) Highly Cross-Adsorbed Secondary Antibody, Alexa Fluor 555: https://www.thermofisher.com/antibody/product/Donkey-anti-Mouse-IgG-H-L-Highly-Cross-Adsorbed-Secondary-Antibody-Polyclonal/A-31570
Goat anti-Rat IgG (H+L) Cross-Adsorbed Secondary Antibody, Alexa Fluor 488: https://www.thermofisher.com/antibody/product/Goat-anti-Rat-IgG-H-L-Cross-Adsorbed-Secondary-Antibody-Polyclonal/A-11006
Donkey anti-Rabbit IgG (H+L) Highly Cross-Adsorbed Secondary Antibody, Alexa Fluor 555: https://www.thermofisher.com/antibody/product/Donkey-anti-Rabbit-IgG-H-L-Highly-Cross-Adsorbed-Secondary-Antibody-Polyclonal/A-31572

Eukaryotic cell lines

Policy information about cell lines and Sex and Gender in Research

Cell line source(s)	MLE12 (American Type Culture Collection, Cat. no: CRL-2110)<br>LLC1 (American Type Culture Collection, Cat. no: CRL-1642)<br>BEAS-2B (B2B) (American Type Culture Collection, Cat. no: CRL-3588)<br>A549 (American Type Culture Collection, Cat. no: CCL-185)<br>A427(American Type Culture Collection, Cat. no: HTB-53)<br>H322 (Sigma Aldrich Cat. no: 95111734)<br>H82 (American Type Culture Collection, Cat. no: HTB-175)<br>H69 (American Type Culture Collection, Cat. no: HTB-119)<br>HEK293T (American Type Culture Collection, Cat. no: CRL-3216)
Authentication	MLE12, LLC1, BEAS-2B (B2B), A549, A427, H322, H82, H69 and HEK293T cells were authenticated by ATCC and Sigma

Mycoplasma contamination	Cell lines were mycoplasma negative.
Commonly misidentified lines (See ICLAC register)	No commonly misidentified lines were used in the study.

Animals and other research organisms

Policy information about studies involving animals; ARRIVE guidelines recommended for reporting animal research, and Sex and Gender in Research

Laboratory animals	Mice (C57BL/6J, BALB/c Nude, Rnf20tm1a(EUCOMM)Wtsi on a C57BL/6 background) were housed in a pathogen-free animal facility under standard conditions with a 12 hour light/dark cycle, temperature of 20-25 ℃ and humidity range of 30-70%. All animal experiments were performed according to the institutional guidelines and are covered in an approved animal experimental protocols by the Committee for Animal Rights Protection of the State of Baden-Württemberg (Regierungspraesidium Karlsruhe, Experimental protocol Az.: 35-9185.81/G-260/17; Az.: 35-9185.81/G-119/23).
Wild animals	No wild animals were used in this study.
Reporting on sex	Both male and female mice were used within the study.
Field-collected samples	No field-collected samples were used in this study.
Ethics oversight	Animal experiments with the Rnf20tm1a(EUCOMM)Wtsi mouse model were performed according to the institutional guidelines and were covered in an approved animal experimental protocol by the Committee for Animal Rights Protection of the State of Baden-Württemberg (Regierungspraesidium Karlsruhe, Experimental protocol Az.: 35-9185.81/G-260/17).<br>Animal experiments involving subcutaneous or intravenous injections were conducted in accordance with institutional guidelines and an animal experimental protocol approved by the Committee for Animal Rights Protection of the State of Baden-Württemberg (Regierungspraesidium Karlsruhe, Experimental protocol Az.: 35-9185.81/G-119/23). The C57BL/6J and the BALB/c Nude mouse line was purchased from Janvier Lab and kept under pathogen-free conditions at the Core Facility Preclinical Models of Medical Faculty Mannheim.

Note that full information on the approval of the study protocol must also be provided in the manuscript.

Plants

Seed stocks	*Report on the source of all seed stocks or other plant material used. If applicable, state the seed stock centre and catalogue number. If plant specimens were collected from the field, describe the collection location, date and sampling procedures.*
Novel plant genotypes	*Describe the methods by which all novel plant genotypes were produced. This includes those generated by transgenic approaches, gene editing, chemical/radiation-based mutagenesis and hybridization. For transgenic lines, describe the transformation method, the number of independent lines analyzed and the generation upon which experiments were performed. For gene-edited lines, describe the editor used, the endogenous sequence targeted for editing, the targeting guide RNA sequence (if applicable) and how the editor was applied.*
Authentication	*Describe any authentication procedures for each seed stock used or novel genotype generated. Describe any experiments used to assess the effect of a mutation and, where applicable, how potential secondary effects (e.g. second site T-DNA insertions, mosiacism, off-target gene editing) were examined.*

ChIP-seq

Data deposition

☒ Confirm that both raw and final processed data have been deposited in a public database such as GEO.

☒ Confirm that you have deposited or provided access to graph files (e.g. BED files) for the called peaks.

Data access links<br>*May remain private before publication.*	Accession number: GSE231317
Files in database submission	GSM7256979 MLE12_Rnf20_Ctrl_RNA_seq_Rep1<br>GSM7256980 MLE12_Rnf20_Ctrl_RNA_seq_Rep2<br>GSM7256981 MLE12_Rnf20_Ctrl_RNA_seq_Rep3<br>GSM7256982 MLE12_Rnf20_Het_RNA_seq_Rep1<br>GSM7256983 MLE12_Rnf20_Het_RNA_seq_Rep2<br>GSM7256984 MLE12_Rnf20_Het_RNA_seq_Rep3<br>GSM7256985 MLE12_Rnf20_CTR_Pol2_chipseq_IP_rep1<br>GSM7256986 MLE12_Rnf20_CTR_Pol2_chipseq_IP_rep2<br>GSM7256987 MLE12_Rnf20_CTR_Pol2_chipseq_IP_rep3<br>GSM7256988 MLE12_Rnf20_HET_Pol2_chipseq_IP_rep1<br>GSM7256989 MLE12_Rnf20_HET_Pol2_chipseq_IP_rep2<br>GSM7256990 MLE12_Rnf20_HET_Pol2_chipseq_IP_rep3

GSM7256991 MLE12_Rnf20_CTR_Pol2_chipseq_INPUT_rep1
GSM7256992 MLE12_Rnf20_CTR_Pol2_chipseq_INPUT_rep2
GSM7256993 MLE12_Rnf20_CTR_Pol2_chipseq_INPUT_rep3
GSM7256994 MLE12_Rnf20_HET_Pol2_chipseq_INPUT_rep1
GSM7256995 MLE12_Rnf20_HET_Pol2_chipseq_INPUT_rep2
GSM7256996 MLE12_Rnf20_HET_Pol2_chipseq_INPUT_rep3
GSM7256997 MLE12_Rnf20_CTR_PLKO_Pol2_chipseq_IP
GSM7256998 MLE12_Rnf20_Het_PLKO_Pol2_chipseq_IP
GSM7256999 MLE12_Rnf20_Het_shHIF1A_Pol2_chipseq_IP
GSM7257000 MLE12_Rnf20_CTR_PLKO_Pol2_chipseq_INPUT
GSM7257001 MLE12_Rnf20_Het_PLKO_Pol2_chipseq_INPUT
GSM7257002 MLE12_Rnf20_Het_shHIF1A_Pol2_chipseq_INPUT
GSM7257003 MLE12_H2Bub_IP_CTR_Rep1
GSM7257004 MLE12_H2Bub_IP_CTR_Rep2
GSM7257005 MLE12_H2Bub_IP_hetRnf20_Rep1
GSM7257006 MLE12_H2Bub_IP_hetRnf20_Rep2
GSM8756871 MLE12_RNF20_HET_H3K4me3_ChIP_r1
GSM8756872 MLE12_RNF20_HET_H3K4me3_ChIP_r2
GSM8756873 MLE12_RNF20_ HET_shHif1a_H3K4me3_ChIP_r1
GSM8756874 MLE12_RNF20_HET_shHif1a_H3K4me3_ChIP_r2
GSM8756875 MLE12_RNF20_HET_H3K4me3_input_r1
GSM8756876 MLE12_RNF20_HET_H3K4me3_input_r2
GSM8756877 MLE12_RNF20_ HET_shHif1a_H3K4me3_input_r1
GSM8756878 MLE12_RNF20_ HET_shHif1a_H3K4me3_input_r2

Genome browser session
(e.g. UCSC)

Provide a link to an anonymized genome browser session for "Initial submission" and "Revised version" documents only, to enable peer review. Write "no longer applicable" for "Final submission" documents.

Methodology

Replicates

RNA_seq in of Ctr and Rnf20_het MLE12 cells
Pol2 ChIP-seq of Ctr and Rnf20_het MLE12 cells
Pol2 ChIP-seq of Ctr_PLKO, Rnf20_het MLE12 cells and Rnf20_het MLE12 cells after shHIF1a.
H2Bub1 ChIP-seq of Ctr and Rnf20_het MLE12 cells.
H3K4me3 ChIP-seq of Rnf20_het MLE12 cells and Rnf20_het MLE12 cells after shHIF1a

Sequencing depth

MLE12_Rnf20_Ctrl_RNA_seq_Rep1.fq.gz 25554900
MLE12_Rnf20_Ctrl_RNA_seq_Rep2.fq.gz 25532555
MLE12_Rnf20_Ctrl_RNA_seq_Rep3.fq.gz 25531926
MLE12_Rnf20_Het_RNA_seq_Rep1.fq.gz 25513990
MLE12_Rnf20_Het_RNA_seq_Rep2.fq.gz 25503582
MLE12_Rnf20_Het_RNA_seq_Rep3.fq.gz 25548401
MLE12_Rnf20_CTR_Pol2_chipseq_IP_rep1_1.fq.gz 17743407
MLE12_Rnf20_CTR_Pol2_chipseq_IP_rep1_2.fq.gz 17743407
MLE12_Rnf20_CTR_Pol2_chipseq_IP_rep2_1.fq.gz 15504173
MLE12_Rnf20_CTR_Pol2_chipseq_IP_rep2_2.fq.gz 15504173
MLE12_Rnf20_CTR_Pol2_chipseq_IP_rep3_1.fq.gz 21424479
MLE12_Rnf20_CTR_Pol2_chipseq_IP_rep3_2.fq.gz 21424479
MLE12_Rnf20_HET_Pol2_chipseq_IP_rep1_1.fq.gz 17768871
MLE12_Rnf20_HET_Pol2_chipseq_IP_rep1_2.fq.gz 17768871
MLE12_Rnf20_HET_Pol2_chipseq_IP_rep2_1.fq.gz 21755498
MLE12_Rnf20_HET_Pol2_chipseq_IP_rep2_2.fq.gz 21755498
MLE12_Rnf20_HET_Pol2_chipseq_IP_rep3_1.fq.gz 21760619
MLE12_Rnf20_HET_Pol2_chipseq_IP_rep3_2.fq.gz 21760619
MLE12_Rnf20_CTR_Pol2_chipseq_INPUT_rep1_1.fq.gz 19527633
MLE12_Rnf20_CTR_Pol2_chipseq_INPUT_rep1_2.fq.gz 19527633
MLE12_Rnf20_CTR_Pol2_chipseq_INPUT_rep2_1.fq.gz 12382347
MLE12_Rnf20_CTR_Pol2_chipseq_INPUT_rep2_2.fq.gz 12382347
MLE12_Rnf20_CTR_Pol2_chipseq_INPUT_rep3_1.fq.gz 20529494
MLE12_Rnf20_CTR_Pol2_chipseq_INPUT_rep3_2.fq.gz 20529494
MLE12_Rnf20_HET_Pol2_chipseq_INPUT_rep1_1.fq.gz 24037605
MLE12_Rnf20_HET_Pol2_chipseq_INPUT_rep1_2.fq.gz 24037605
MLE12_Rnf20_HET_Pol2_chipseq_INPUT_rep2_1.fq.gz 18410683
MLE12_Rnf20_HET_Pol2_chipseq_INPUT_rep2_2.fq.gz 18410683
MLE12_Rnf20_HET_Pol2_chipseq_INPUT_rep3_1.fq.gz 19977456
MLE12_Rnf20_HET_Pol2_chipseq_INPUT_rep3_2.fq.gz 19977456
MLE12_Rnf20_CTR_PLKO_Pol2_chipseq_IP_1.fq.gz 72258399
MLE12_Rnf20_CTR_PLKO_Pol2_chipseq_IP_2.fq.gz 72258399

MLE12_Rnf20_Het_PLKO_Pol2_chipseq_IP_1.fq.gz 30324628
MLE12_Rnf20_Het_PLKO_Pol2_chipseq_IP_2.fq.gz 30324628
MLE12_Rnf20_Het_shHIF1A_Pol2_chipseq_IP_1.fq.gz 57096797
MLE12_Rnf20_Het_shHIF1A_Pol2_chipseq_IP_2.fq.gz 57096797
MLE12_Rnf20_CTR_PLKO_Pol2_chipseq_INPUT_1.fq.gz 73533084
MLE12_Rnf20_CTR_PLKO_Pol2_chipseq_INPUT_2.fq.gz 73533084
MLE12_Rnf20_Het_PLKO_Pol2_chipseq_INPUT_1.fq.gz 6098602
MLE12_Rnf20_Het_PLKO_Pol2_chipseq_INPUT_2.fq.gz 6098602
MLE12_Rnf20_Het_shHIF1A_Pol2_chipseq_INPUT_1.fq.gz 33479619
MLE12_Rnf20_Het_shHIF1A_Pol2_chipseq_INPUT_2.fq.gz 33479619
MLE12_H2Bub_IP_CTR_Rep1.fq.gz 31340004
MLE12_H2Bub_IP_CTR_Rep2.fq.gz 31231521
MLE12_H2Bub_IP_hetRnf20_Rep1.fq.gz 30898523
MLE12_H2Bub_IP_hetRnf20_Rep2.fq.gz 31163882
MLE12_RNF20_HET_H3K4me3_ChIP_r1.fq.gz 18 214 866
MLE12_RNF20_HET_H3K4me3_ChIP_r2.fq.gz 33 260 258
MLE12_RNF20_ HET_shHif1a_H3K4me3_ChIP_r1.fq.gz 19232965
MLE12_RNF20_HET_shHif1a_H3K4me3_ChIP_r2.fq.gz 22 068 607
MLE12_RNF20_HET_H3K4me3_input_r1.fq.gz 18 746 929
MLE12_RNF20_HET_H3K4me3_input_r2.fq.gz 29 937 736
MLE12_RNF20_ HET_shHif1a_H3K4me3_input_r1.fq.gz 28 250 838
MLE12_RNF20_ HET_shHif1a_H3K4me3_input_r2.fq.gz 30 588 376

Antibodies

Pol II antibody Rpb1 NTD (D8L4Y) Cell Signalling, H2Bub1 antibody (ubiquityl Histone H2B, 5546S) Cell Signalling, H3K4me3 (ab8580) Abcam

Peak calling parameters

The pausing index was calculated by dividing the normalized count per million reads (CPM) on the TSS area (-50 to 300bp) by the CPM on the gene body plus 3kb after the transcription termination site (TTS). For the calculation, the GitHub repository code (https://github.com/MiMiroot/PIC) was used with settings mm10.gtf --TSSup 50 --TSSdown 300 --GBdown 3000) and ENSEMBL mm10, version 108.

The t-test from the package rstatix was used to calculate the differential PI values from total Pol II ChIP-Seq of Rnf20+/- versus control MLE12 cells.

Data quality

FastQC

Software

Software for NGS: Bowtie2 version 2.4.4, STAR version 2.7.3a, ngsplot version 2.47.1, Deseq2 version 1.40.0
